# Advances in monitoring and control of refolding kinetics combining PAT and modeling

**DOI:** 10.1007/s00253-021-11151-y

**Published:** 2021-02-17

**Authors:** Jan Niklas Pauk, Janani Raju Palanisamy, Julian Kager, Krisztina Koczka, Gerald Berghammer, Christoph Herwig, Lukas Veiter

**Affiliations:** 1 Research Area Biochemical Engineering, Institute of Chemical, Environmental and Bioscience Engineering, Vienna University of Technology, Gumpendorferstrasse 1a/166, 1060 Vienna, Austria; 2 Competence Center CHASE GmbH, Altenbergerstraße 69, 4040 Linz, Austria; 3 Bilfinger Industrietechnik Salzburg GmbH, Mooslackengasse 17, 1190 Vienna, Austria

**Keywords:** Inclusion body, Protein refolding, M^3^C methodology, Process Analytical technology (PAT), Quality by Design (QbD), Model Predictive Control (MPC)

## Abstract

**Abstract:**

Overexpression of recombinant proteins in *Escherichia coli* results in misfolded and non-active protein aggregates in the cytoplasm, so-called inclusion bodies (IB). In recent years, a change in the mindset regarding IBs could be observed: IBs are no longer considered an unwanted waste product, but a valid alternative to produce a product with high yield, purity, and stability in short process times. However, solubilization of IBs and subsequent refolding is necessary to obtain a correctly folded and active product. This protein refolding process is a crucial downstream unit operation—commonly done as a dilution in batch or fed-batch mode. Drawbacks of the state-of-the-art include the following: the large volume of buffers and capacities of refolding tanks, issues with uniform mixing, challenging analytics at low protein concentrations, reaction kinetics in non-usable aggregates, and generally low re-folding yields. There is no generic platform procedure available and a lack of robust control strategies. The introduction of Quality by Design (QbD) is the method-of-choice to provide a controlled and reproducible refolding environment. However, reliable online monitoring techniques to describe the refolding kinetics in real-time are scarce. In our view, only monitoring and control of re-folding kinetics can ensure a productive, scalable, and versatile platform technology for re-folding processes. For this review, we screened the current literature for a combination of online process analytical technology (PAT) and modeling techniques to ensure a controlled refolding process. Based on our research, we propose an integrated approach based on the idea that all aspects that cannot be monitored directly are estimated via digital twins and used in real-time for process control.

**Key points:**

*• Monitoring and a thorough understanding of refolding kinetics are essential for model-based control of refolding processes.*

*• The introduction of Quality by Design combining Process Analytical Technology and modeling ensures a robust platform for inclusion body refolding.*

## Introduction

Overexpression of proteins in *Escherichia coli* results in inclusion bodies (IBs) which generally are biologically inactive and need to be processed to yield an active, final product. However, this disadvantage has to be considered in view of several benefits of IBs like high purity, simple separation from cells and stability against mechanical, thermal, and proteolytic stress (Humer and Spadiut 2018). The native structure of proteins is recovered from IBs via solubilization followed by the refolding process. Solubilization is performed with denaturants in the presence of reducing agents. After the IBs have been solubilized, protein refolding can be achieved by decreasing the amount of denaturant and providing a suitable environment for the protein to refold into its native structure (Jungbauer and Kaar 2007). This is a critical step toward the efficient recovery of proteins (Yamaguchi and Miyazaki 2014). During refolding, the denatured solubilized protein first forms its secondary structure as part of a self-folding process, which further leads to a final stable native tertiary or quaternary structure, depending on the protein. However, transient intermediates formed during the process may engage in non-specific intermolecular interactions—primarily due to their exposed hydrophobic surfaces—resulting in aggregates and a major loss of yield (Mayer and Buchner 2004; Su et al. 2011). For simplification, a reduced view of this refolding process is useful consisting of only three distinct protein forms: solubilized protein S as the starting material for the refolding process, native protein N as the final product in a correct, and active form and aggregated protein A consisting of all folded but not active proteins including folding intermediates. These three defined protein forms will be referenced over the course of this review.

Numerous refolding methods are available such as dilution, dialysis, diafiltration, on-column refolding, or refolding by high hydrostatic pressure (Jungbauer and Kaar 2007; Middelberg 2002; Singh et al. 2015; Yamaguchi and Miyazaki 2014; Qoronfleh et al. 2007). Other approaches add additives to the refolding buffer to increase the refolding yield such as arginine or polyethylene glycol (Cleland et al. 1992; Kudou et al. 2011; Rathore et al. 2013). A recent and detailed summary of appropriate solubilization and refolding procedures can be found in the publication by Singhvi et al. (Singhvi et al. 2020). All these techniques aim to achieve a higher refolding efficiency while avoiding aggregate formation. Dilution is the traditional approach most widely applied on an industrial level (Jungbauer and Kaar 2007; Singh et al. 2015). In commercial applications, dialysis is rather time-consuming as it depends on the slow diffusion of ions and molecules. In addition, this may result in aggregate formation due to prolonged protein exposure at medium denaturant concentration (Cabrita and Bottomley 2004; Tsumoto et al. 2003). Diafiltration is shown to be an efficient system for protein refolding, not only to achieve higher yields than refolding in batch mode, but by reducing buffer consumption as well (Ryś et al. 2015a). However, fouling of membranes by aggregated proteins poses a problem (De Bernardez Clark 1998; Ryś et al. 2015a). The applied pressure in the high-pressure refolding method dissociates the existing protein aggregates and prevents the formation of further aggregates while requiring only small concentrations of chaotropic substances. Thus, this method can combine protein solubilization and refolding in one operation and is in most cases not restricted to low protein concentrations (Qoronfleh et al. 2007).

Despite the advantages of the aforementioned alternatives, the industry seems to be reluctant to abandon the extensively studied and well-established dilution method for protein refolding and thus it is also the main focus of this review. As part of this procedure, the solubilized IBs are mixed with refolding buffer at large volumes typically resulting in a 10- to 50-fold dilution of denaturants and a final protein concentration of 1–100 μg/mL (Pathak et al. 2016). From an industrial perspective, the method’s simplicity, suitability for screening of additives, and easy implementation at various scales (Mirhosseini et al. 2019; Su et al. 2011) are advantageous. Also, refolding kinetics can be influenced independently of other effects, unlike during on-column refolding or diafiltration, which is an important aspect in view of the current industrial trend of increased process control. However, further processing of the refolded proteins requires concentration steps since the handling of low protein concentrations and high volumes is difficult and time intensive (Rathore et al. 2013; Singh and Panda 2005; Su et al. 2011). The dilution can be performed quickly and efficiently in two ways. As for the “dilution by batch mode,” the solubilized IBs can be added directly to the refolding buffer as a single batch, which allows the denaturants and solubilized proteins to be diluted in a short time. However, this approach poses a higher risk of aggregation and misfolding due to the inefficient mixing at large reaction volumes and the formation of protein concentration centers while the solubilized protein is forced to reach its native structure quickly. The more effective alternative, “dilution by fed-batch mode,” is to slowly dilute the solubilized IBs by gradually adding them to the refolding mixture either drop-wise, in a pulsed way or as a constant, fed-batch-like addition (Cabrita and Bottomley 2004; Katoh and Katoh 2000). This method provides the denatured proteins with enough time for folding and thus prevents the forming of aggregations in the early folding pathway (De Bernardez Clark 1998).

For an efficient refolding process with the dilution by fed-batch mode, several parameters need to be considered such as denaturant concentration (De Bernardez Clark 1998; Dong et al. 2004; Tsumoto et al. 2003), protein concentration, mixing intensity, reaction temperature, and buffer components (Anselment et al. 2010; Eiberle and Jungbauer 2010) as well as dissolved oxygen and redox potential for proteins with disulfide bonds (Pizarro et al. 2009). Furthermore, the process needs to be adequately monitored to achieve good product quality. Consequently, possible measurement techniques are needed to assure process monitoring and product quality, as stated by the authorities in the PAT and QbD approaches (Food and Drug Administration 2004). The supporting use of modeling and simulation techniques in dilution refolding is regarded as a potent method to overcome the previously mentioned limitations of the process as well as limited available measurements (Dong et al. 2004; Jungbauer and Kaar 2007; Kiefhaber et al. 1991) and to generate platform knowledge applicable for a range of IB products (Humer and Spadiut 2018). Full-state estimation in the form of a digital twin, e.g., through Kalman or particle filters, allows the control of the process in real time and thus would satisfy the industrial need for product quality assurance and process optimization besides providing a fault detection system.

In this review, we focus on the measurement, modeling, monitoring, and control of protein refolding processes to achieve optimal model-based control. The first section “Analytical methods of refolding processes” focuses on the timely assessment of refolding yield during dilution refolding, and “Models of refolding processes” describes current refolding models in the literature; in “Monitoring of refolding processes” and “Control of refolding processes” we will describe computational methods to control refolding kinetics in real time using modeling techniques including soft sensors and model-based control. Finally, we summarize our findings under “Proposal for a model-based refolding control strategy” including a detailed proposal for controlled kinetics of dilution refolding combining PAT and modeling according to the industrial need in agreement with the QbD guidelines.

## Analytical methods of refolding processes

To successfully monitor refolding kinetics in a dilution refolding process, three distinct refolding species need to be analyzed timely and with high specificity: solubilized IBs, refolded protein, and protein aggregates. In the following, several analytical methods suitable for the monitoring of these aspects are discussed. Each method is applicable from the perspective of dilution refolding processes. For each technique, the general measurement procedure and the potential gain of information are covered, corresponding advantages and disadvantages are characterized as well. Finally, a summary of the recommended use and potential benefits of these methods is given.

### Circular dichroism

Circular dichroism (CD) is used for the analysis of protein folding on the basis that folded and unfolded proteins show different spectra (Greenfield 2006). To study a dilution refolding process, a stopped-flow instrument with high-mixing capability can be combined with CD for online spectral analysis. As part of this method, the protein sample is first mixed with a denaturant to make it unfold, which is followed by a dilution step with the required buffer. Thus, with a low concentration of denaturant in the system, refolding may start and can be monitored by CD (Clarke 2012). In general, secondary structure information of proteins is obtained from the far UV range of their spectrum, as amide bonds absorb light at 180–250 nm. Additionally, the aromatic amino acids and disulfide bonds provide information on the tertiary structure in the near UV ranges (250–320 nm) (Lindon et al. 2016).

The optimal protein concentration of a sample for CD analysis ranges from 0.2 to 1 mg/mL, depending on the path length of the applied cell that is normally between 0.01 and 0.05 cm (Greenfield 2006). The sample preparation involves a filtering step at 0.2 μm to obtain a homogenous solution that is free from scattering particles. Additionally, it is generally recommended to avoid a high concentration of chloride ions in sample buffers since they absorb strongly at wavelengths below 195 nm (Pelton and McLean 2000).

CD is a highly sensitive, robust, and non-destructive method suitable for the study of protein folding. Analysis can be carried out in a wide range of solvent environments with very small quantities of liquid (Kelly et al. 2005; Micsonai et al. 2015). However, optimal protein concentration ranges are rather high for refolding purposes (0.2 to 1.0 mg/mL) and a feedback time within minutes might be too long for process control, depending on data analysis of different spectra (Clarke 2012). Also, the nature of the transient refolding intermediates cannot be predicted completely using this technique.

### Fluorescence spectroscopy

In fluorescence spectroscopy, the fluorescence intensity measured as a function of a wavelength is recorded during each stage of the refolding process to predict the conformations and structure of proteins (Printz and Friess 2012). Offline analysis can be conducted in a stopped-flow system where the reactants are swiftly mixed in a cuvette, while the change in fluorescence intensity is monitored over time (Ladokhin 2009; Lew et al. 1997; Qin and Pyle 1997).

Protein folding studies by fluorescence spectroscopy are based on the monitoring of fluorescence originating from fluorophores in the sample. Once the protein folds to form its tertiary structure, some of the fluorophores become covered in the inner hydrophobic environment resulting in high quantum yield and hence large fluorescence intensity. In comparison, in a partially folded or unfolded state, these compounds are exposed to the hydrophilic environment of the solvent leading to low fluorescence intensity. Hydrophobic interactions thus help to determine the conformation, solubility, or aggregation properties of a protein (Lamba et al. 2009). In case of intrinsic fluorescence, the signal originates from the naturally occurring aromatic amino acid residues tryptophan and tyrosine present in the sample (Moore-Kelly et al. 2019). Tryptophan and tyrosine are usually buried within the protein core in the native folded state and only get exposed to the hydrophilic environment of the solvent during a partially folded or unfolded state (Lakowicz 1988). However, when such intrinsic fluorophores are not present in the sample, an extrinsic fluorescence signal can be generated by the covalent attachment of dyes to the protein in order to be able to monitor subtle changes in hydrophobicity. Dyes such as ANS (1-anilinonaphthalene-8-sulfonate) and Nile red have been used in refolding experiments for monitoring differences in surface hydrophobicity and for the detection of aggregates (Hawe et al. 2008; Pathak et al. 2016; Sutter et al. 2007).

Fluorescence spectroscopy is a sensitive method offering rapid data acquisition and sample analysis even at sub-nanomolar concentrations with a low feedback time within seconds (Jazaj et al. 2019; Ladokhin 2009). An optimal performance of the measurements is largely dependent on the selection of a suitable excitation wavelength according to the fluorophore. Optimal emission wavelengths need to be evaluated by a wavelength scan between 310 and 450 nm for tryptophan fluorescence, 400–600 nm for ANS, and 565–750 nm for Nile red (Hawe et al. 2008; Lamba et al. 2009). Drawbacks of this method include intrinsic fluorophores being limited to proteins containing tryptophan and tyrosine residues, while extrinsic fluorophores may alter not only the stability of the proteins but also the folding kinetics due to their covalent attachment (Pathak et al. 2016). Therefore, the use of extrinsic fluorophores might interfere with the refolding process itself making its use for process control potentially problematic.

### Infrared spectroscopy

Fourier-transform infrared spectroscopy (FTIR) can be applied to predict the secondary structure of proteins by analyzing which wavelengths of radiation in the infrared region of the spectrum are absorbed by the sample (Tatulian 2019). Thereby, Fourier transformation enables the decomposition of a detector obtained time-domain spectrum into its constituent frequency domain spectra that can be easily interpreted (Griffiths 1983). With the inline use of a fiber active attenuated total reflection (ATR) probe, online analysis is possible during dilution refolding processes with high sensitivity and in real time. Offline monitoring is performed by manual sampling and analyzing of samples in a cuvette (Walther et al. 2014).

FTIR can illuminate the structural related changes during the refolding process and can be used to determine when to terminate the process to avoid protein loss due to aggregation. In comparison to CD, salt solutions are not problematic and turbid samples can be analyzed (Gregoire et al. 2012; Pelton and McLean 2000; Walther et al. 2014). Further advantages include low feedback time (within seconds) using online sample analysis, which resolves reproducibility problems that arise from offline sample preparation by other methods. Additionally, water can be used for solvent preparation since it gets eliminated as background noise in the resulting spectra. The minimum sample concentration for this method is 0.01 g/L (Humer and Spadiut 2018). The downside of ATR-FTIR is the high sensitivity of probes to vibrations (Schuttlefield and Grassian 2008) and interference with common solubilization buffer components like urea or guanidinium chloride (Hauser 2013), as well as IR absorbance of water in the same range as proteins (Pathak et al. 2016).

### Raman spectroscopy

Raman spectroscopy is used to analyze changes in the secondary structure of proteins (Brewster et al. 2013) and the formation of disulfide bonds (Wang et al. 2016). Traditional Raman spectroscopy is used to study protein solubilization (Brewster et al. 2013) and aggregation (Dolui et al. 2020) at protein concentrations of 1 g/L (Wen 2007). The method is very sensitive to small conformational changes (Brewster et al. 2013), is non-destructive, needs low to no sample preparation, and is insensitive to water (Bunaciu et al. 2015). However, solubilization and refolding buffer components show on the spectra and have to be subtracted by performing a reference analysis of the buffers (Brewster et al. 2013), especially with changing concentrations due to the refolding with dilution by fed-batch mode. Therefore, it might be applied for real-time monitoring of refolding processes.

Innovative alterations such as the surface-enhanced resonance Raman spectroscopy (SERS) enable measurements of concentrations as low as 0.08 g/L at feedback times of 5 min (Eryilmaz et al. 2017), whereas time-resolved resonance Raman spectroscopy (TR RR) allows the monitoring of solubilization and refolding of proteins at time resolutions of down to 100 μs (Buhrke and Hildebrandt 2020). However, both techniques are not suitable for real-time monitoring of a protein refolding process, because SERS requires the analyte to be adsorbed onto metal particles (Eryilmaz et al. 2017) and TR RR requires chromophores such as aromatic amino acids or cofactors (Buhrke and Hildebrandt 2020). Thus, the TR RR analysis is restricted to specific proteins and often reflects only local parts of these proteins (Buhrke and Hildebrandt 2020).

### Nuclear magnetic resonance spectroscopy

In protein analysis, nuclear magnetic resonance spectroscopy (NMR) provides information on the molecular and geometric composition of covalent bonds, while additionally enabling assessment of non-covalent bonds between neighboring atoms (Wüthrich 2003). In refolding, this technique has been used to determine secondary structure in inclusion bodies (Umetsu et al. 2004), the structure of transient protein configurations during refolding (Dyson and Wright 2004), and more generally the formation of a protein’s native, tertiary structure over time (Humer and Spadiut 2018; Ogura et al. 2013). NMR spectra of refolded protein samples at different refolding times in the form of chemical shifts may indicate completion of refolding processes after formation of native protein structures (Pathak et al. 2016).

NMR is not suitable for routine real-time monitoring of refolding processes, as it requires protein purification and concentration prior to measurement (Ogura et al. 2013); the minimum sample concentration is rather high in the range of 0.1–3 mM (Kelly et al. 2005); additionally, the method is limited to protein sizes below 40–70 kDa (Frueh et al. 2013).

### Light scattering techniques

Dynamic light scattering (DLS) is a measurement technique applicable in real time during refolding processes to monitor protein refolding and aggregation. Fluctuations in light scattering are detected to determine the hydrodynamic size of the particles (Yu et al. 2013). The radius of the protein is proportional to its folding state; thus, the smallest radius of the protein represents its completely folded form, while the largest corresponds to the unfolded state or denatured state. DLS detects aggregates based on the fact that the size of native proteins and aggregates varies considerably (Amin et al. 2014; Yasuda et al. 1998; Yu et al. 2013). A cuvette-based method can be used in an online capacity combining the measuring cell with automated sampling, similar to online flow cytometry approaches (Veiter and Herwig 2019). As an alternative to cuvette-based measurements, an integrated fiber optic probe can also be used. This approach has enabled online analysis of samples (Dhadwal et al. 1993).

With DLS, a sample range of 0.1–50 mg/mL can be estimated at a range of diameters from 1–2 nm to 3–5 μm. DLS is a fast and non-destructive technique, where quantification can be completed within a minute of sampling and samples can be re-used. This method has advantages over spectroscopic techniques, as it does not require a correlation between secondary and tertiary structures as in CD spectroscopy and it can be applied to all proteins, as there is no need for intrinsic and extrinsic fluorophores as in fluorescence spectroscopy (Dhadwal et al. 1993; Yu et al. 2013).

A drawback of this analysis is poor resolution due to a limited differentiation between particle species. Furthermore, the method is sensitive to the presence of particles in the refolding buffer, but this can be addressed by filtering the samples. Overall, DLS is a qualitative tool and not a quantitative one; however, Yu et al. showed a good correlation between DLS and SEC aggregation data which enabled quantitative statements (Den Engelsman et al. 2011; Yu et al. 2013).

Another powerful light scattering–based technique is multi-angle light scattering (MALS) which increases the robustness of the measurement by measuring the scattered light at multiple angles simultaneously and, thus, preventing to omit populations present in the sample (Naiim et al. 2015). This method can be used in combination with size exclusion chromatography or more recently ion exchange chromatography (Amartely et al. 2018) enabling the determination of molar masses of peaks separated by the chromatographic steps. Protein shape, aggregation, and oligomerization can be characterized as described by Machuca and Roujeinikova (2017) and Hemmig et al. (2005).

### Reversed-phase HPLC

During the refolding process, chromatographic techniques can be used for the separation and quantification of folded and unfolded protein species to identify the current extent of refolding. Online applicability of this technique is possible using automated sampling and sample processing. For this purpose, sampling and potentially dilution need to be performed in a modular PAT system with a connected HPLC (Veiter and Herwig 2019). Samples need to be collected at different time points and analyzed based on the hydrophobicity of the respective proteins by reversed-phase HPLC (RP-HPLC) with an UV detector. As reduced proteins are completely unfolded resulting in an open structure, their hydrophobicity will be greater compared to that of native and oxidized (incorrect disulfide bondings) proteins (Cho et al. 2001; Choi et al. 2005; Pathak et al. 2016). If oxidized impurities occur, they typically display lower hydrophobicity than the native protein due to the differences in the disulfide linkages (Pathak et al. 2016; Rathore et al. 2013). However, different states of proteins not containing disulfide bonds cannot be separated effectively, because high temperatures of the method can destabilize the protein structure and consequently lead to unfolding of all protein states during the measurement (Pathak et al. 2016).

A robust RP-HPLC method is a powerful protein quantification system, with a limit of quantification (LOQ) lower than 10 μg/mL. The minimum sample concentration is 0.3 g/L assuming a sample volume of 2 μL. However, RP-HPLC is a time-consuming technique that can take 20–80 min for the high-resolution analysis of a sample. Consequently, a timely depiction of refolding kinetics can be problematic. Furthermore, overloading should be avoided by applying less than 1 mg per 1 mL of the column to have a better resolution of the resulting peaks (Humer and Spadiut 2018; Lindon et al. 2016; Živančev et al. 2015). Moreover, higher temperature during analysis improves recovery and plays a key role in selectivity and resolution, but it might induce aggregation of proteins during analysis (Hussain et al. 2019).

A major drawback of this method is the relatively large feedback time (several minutes) even when using a sampling device, such that other measurements are needed as information sources concerning monitoring and control of the process.

### Size-exclusion HPLC

Further processing of samples using size-exclusion HPLC (SEC-HPLC) facilitates the monitoring of aggregates during refolding. Thereby, a timely depiction of the changes in aggregate kinetics is possible (Choi et al. 2005; Pathak et al. 2016). SEC-HPLC separation is based on size, enabling the isolation of oligomeric aggregates. The native, correctly folded proteins have a more compact shape and size; therefore, they are distinguishable from unfolded and partially folded proteins (Cowan et al. 2008; Davidson 2008). Due to the flexibility and reproducibility of the process, SEC-HPLC is considered the standard process for measuring the aggregation of proteins (Hong et al. 2012).

In order to maintain optimal resolution and sensitivity, an automated sampling device—as previously mentioned—needs to control the sample volume, ideally as 5–10% of the total volume of the column (Cowan et al. 2008). Using an UV detector, lower wavelengths (214 or 220 nm) are optimal for the high sensitivity measurement of proteins present in low concentrations, while higher wavelengths (280 nm) enable the linear range detection of major species. With the dual-wavelength detection method, two wavelength ranges can be obtained. The wavelength ratio, which is the ratio of absorbance from two wavelengths, helps in the high sensitivity determination of the aggregate percentage (Hong et al. 2012; Printz and Friess 2012). Next to a UV detector, a fluorescence detector may also be used to enhance the selectivity and sensitivity of the method, and in general to further facilitate the measurement and quantification of protein content (Hong et al. 2012).

SEC-HPLC is a robust and sensitive analytical technique. Because of its high reproducibility and flexibility, it is a common approach for quantitative analysis of proteins (Amin et al. 2014; Hong et al. 2012). Potential drawbacks of the method include non-ideal interactions between large molecules and column packing materials, which might negatively affect the retention time, recovery, and peak shape (Hong et al. 2012). Similar to RP-HPLC, this method has long feedback times and is therefore problematic for monitoring and control of the process.

### Summarizing the use of the presented analytical methods

Since the refolding of proteins is on a timescale of minutes to hours, real-time monitoring tools are preferred (Glassey et al. 2011). Some of the methods discussed in this section provide information on key aspects of the refolding process itself; Table 1 summarizes the characteristics of these techniques including information on concentration ranges, feedback time, and unwanted interaction with buffer components. CD and fluorescence spectroscopy are complementary spectral analysis techniques. With CD spectroscopy, the conformational changes of proteins during the refolding process can be followed, while fluorescence spectroscopy detects changes of the aromatic residues in the protein backbone (Reed et al. 2014). FTIR spectroscopy can resolve the limitations of CD spectroscopy regarding turbid and high-salt samples and has advantages over fluorescence spectroscopy as additional sample preparations can be avoided. Furthermore, FTIR can be applied for online monitoring of refolding processes (Walther et al. 2014).

**Table 1:** Summary of the analytical methods discussed in this section. Online analysis is defined as automated sampling connected to the process followed by timely evaluation. Offline analysis is defined as manual sampling, typically followed by discontinuous sample preparation, measurement, and evaluation

Techniques	Type of analysis	Target measurement	Concentration range	Advantages	Disadvantages	References
CD	Online Offline	Folded/unfolded protein and intermediates: secondary structure, tertiary structure	Optimal range: 0.2 to 1.0 mg/mL Far UV-CD: 0.25 mg/mL Near UV-CD: 2.5 mg/mL	• Highly sensitive • Reliable • Accurate • Non-destructive • Wide range of solvent environments can be used	• No prediction of transient intermediates • No information on residues • High protein concentration needed, problematic for refolding processes • Feedback/data analysis time (several minutes) of CD spectra might be too long for process control • Interference with buffers with high ionic strength	(Clarke 2012) (Greenfield 2006) (Kelly et al. 2005) (Lindon et al. 2016) (Micsonai et al. 2015) (Pathak et al. 2016)
Fluorescence spectroscopy	Online Offline	Folded/unfolded protein: tertiary structure, partially folded or unfolded state	0.015 mg/mL depending on protein size	• Sensitive • Performed at sub-nanomolar concentrations • Rapid data acquisition • High signal-to-noise ratio • Low feedback time (seconds), applicable for process control	• Intrinsic fluorophores are limited to proteins containing tryptophan and tyrosine residues • Extrinsic fluorophores alter stability of the proteins and affect kinetics of protein folding due to covalent attachment to the protein	(Hawe et al. 2008) (Ladokhin 2009) (Lamba et al. 2009) (Lew et al. 1997) (Pathak et al. 2016)
FTIR	Online Offline	Folded/unfolded protein: structural related changes during refolding	> 0.01 mg/mL	• Use of salt buffers possible • Analyses of turbid samples • Direct sample analysis resolving reproducibility issues • Low feedback time (seconds) applicable for process control	• Probes sensitive to vibrations • Interaction with common solubilization buffer compounds like urea and guanidinium chloride • Changing background buffer signals due to dilution refolding by fed-batch mode	(Pathak et al. 2016) (Pelton and McLean 2000) (Tatulian 2019) (Walther et al. 2014)
Raman spectroscopy	Online Offline	Folded/unfolded protein: secondary structure, disulfide bond formation/tertiary structure	> 1 mg/mL SERS: > 0.08 mg/mL	• Non-destructive • No sample preparation • Insensitive to water • Sensitive to small conformational changes • Very low feedback times (TR RR)	• Interaction with common solubilization buffer compounds like urea and guanidium hydrochloride • Changing background buffer signals due to dilution refolding by fed-batch mode • Feedback times of few minutes • TR RR restricted to proteins containing chromophores	(Brewster et al. 2013) (Buhrke and Hildebrandt 2020) (Bunaciu et al. 2015) (Eryilmaz et al. 2017) (Wang et al. 2016) (Wen 2007)
NMR	Offline	Formation of native, tertiary structure over time	> 1 g/L 0.1–5 mM	• Information on tertiary protein structure	• Protein purification and concentration necessary • Limited to protein sizes below 50 kDa	(Frueh et al. 2013) (Kelly et al. 2005) (Pathak et al. 2016)
DLS MALS	Offline Online	Protein refolding and aggregation: radius of protein is proportional to its folding state, size of aggregates and native protein varies considerably	> 0.05 mg/mL	• Nondestructive • Does not require correlation between secondary and tertiary structure • No need for intrinsic and extrinsic fluorophores • Low feedback time (seconds) applicable for process control	• Primarily a qualitative method • Complicated • Poor resolution • Sensitive to particle presence in refolding buffer	(Amartely et al. 2018) (Amin et al. 2014) (Dhadwal et al. 1993) (Hemmig et al. 2005) (Machuca and Roujeinikova 2017) (Yasuda et al. 1998) (Yu et al. 2013)
RP-HPLC	Online Offline	Solubilized protein and native protein, loss through aggregation can be estimated	> 0.01 mg/mL	• Robust • High resolution • Quantification of solubilized, native and misfolded protein states if the protein contains disulfide bonds	• High temperature induces aggregation of proteins • Only quantification of soluble protein species possible if the protein does not contain disulfide bonds • Feedback time (several minutes) problematic for use in process control • Unwanted effects with components of refolding buffer possible (e.g., PEG)	(Cho et al. 2001) (Choi et al. 2005) (Pathak et al. 2016) (Rathore et al. 2013)
SEC-HPLC	Online Offline	Aggregates and native protein	> 0.01 mg/mL	• High reproducibility • Sensitive • Robust	• Non-ideal interactions between large molecules and column packing materials • Feedback time (several minutes) problematic for use in process control	(Hong et al. 2012) (Pathak et al. 2016) (Printz and Friess 2012)

To quantify the amount of protein and aggregates during refolding, SEC-HPLC and RP-HPLC are commonly used and robust techniques. Analysis can be carried out at multiple sampling points in near real time if an automated sampling device is available (Veiter and Herwig 2019). Also, DLS can be used to evaluate the formation of high-molecular-weight aggregates, but it is mainly used for qualitative measurements (Amin et al. 2014; Den Engelsman et al. 2011). FTIR and fluorescence spectroscopy can also be applied for aggregate monitoring, but the former method is not very sensitive at low aggregate concentrations and the latter might alter the behavior of the sample protein in case the binding of extrinsic fluorophores is necessary (Sutter et al. 2007; Walther et al. 2014).

There is no universal tool that can be applied to understand each aspect of the refolding process. To address this issue, a number of researchers employed a selection of techniques: Umetsu et al. studied secondary structure formation and tertiary structure by CD spectroscopy, explored folding by fluorescence spectroscopy using the shift in tryptophan emission, and performed FTIR for structural analysis of aggregated materials (Umetsu et al. 2003); Vincentelli et al. conducted CD spectroscopy to investigate protein folding and DLS for protein aggregation (Vincentelli et al. 2004); Cowan et al. used CD spectroscopy and SEC-HPLC to analyze protein refolding, and to study the protein’s multimeric state (Cowan et al. 2008); Pathak et al. performed RP-HPLC for disulfide linkage analysis accompanied by SEC-HPLC for the study of aggregates and carried out CD spectroscopy for the investigation of secondary structure (Pathak et al. 2016). To address the problem of long feedback times when using RP-HPLC, Pathak et al. (2016) also performed zeta potential analysis: this technique may be used to monitor initial refolding stages involving primary structure, thereby enabling a relatively low analysis time within minutes.

If the monitoring of refolding kinetics is possible, critical process parameters (CPPs) can be defined. Since different analytical methods differ not only in robustness, sensitivity, and accuracy but also in real-time measurement capabilities, the additional use of modeling is a promising possibility to enhance and align several methods. Some of the monitoring techniques described do not feature sufficiently low feedback times for process control. However, these techniques nevertheless provide process knowledge that can be used in modeling approaches which will be discussed in section “Models of refolding processes.” For instance, solubilized IBs and native protein could be quantified offline through SEC-HPLC and RP-HPLC for model refinement, while the refolding process itself can be monitored in real-time through detecting native protein via FTIR and aggregation through DLS or combinations of the aforementioned methods. Another problem arises for the spectroscopic methods with changing reference spectra due to the feeding of the dilution by fed-batch mode. Therefore, updated measurements of the reference spectrum are necessary.

## Models of refolding processes

A model describes the change of a dynamical system over time. Therefore, it enables predictions of the system behavior into the future with given initial conditions. In the case of refolding processes, a model can be utilized to estimate the targeted product forms from other measurements; hence, it functions as a state observer providing additional indirect measurements of otherwise unmeasurable states. Mechanistic models describe a dynamical system with one or a set of differential equations. They are derived from physical first principles such as mass, momentum, heat, or energy balances (Gernaey et al. 2010; Wechselberger et al. 2013). A white box model completely describes a process with first principles and known parameters (Sohlberg and Jacobsen 2008); thus, for this type of model, no process data is necessary. However, if parameters or the underlying differential equations are unknown, data is needed to develop a model. Unknown model parameters in a protein refolding process are the reaction kinetics, which must be identified by fitting the process model on experimental data.

In the following, representative models for protein refolding and techniques to analyze their applicability are described.

**Fig. 1:**
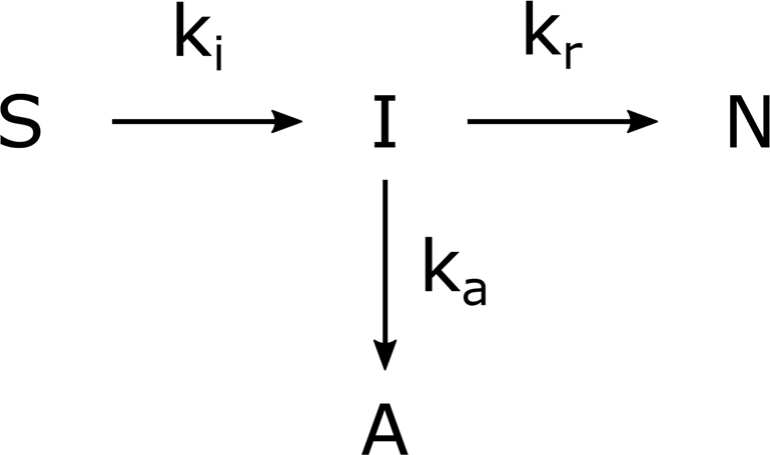
Model depicting a refolding reaction scheme with on-pathway folding intermediate and off-pathway aggregation (Hevehan and de Bernardez Clark 1997). *S* corresponds to the solubilized protein, *I* stands for the folding intermediates, *A* for the protein aggregates and *N* represents the native protein. *k* signifies the corresponding reaction rate

The mechanism behind the folding of proteins is of great interest and many research groups tried to describe it using a variety of models (Cleland et al. 1992; Dong et al. 2004; Jungbauer and Kaar 2007; Kiefhaber et al. 1991; Zettlmeissl et al. 1979; Ryś et al. 2015a). The general structure of these models resembles one of the three classical models shown by Dill and Chan (Dill and Chan 1997), namely

the off-pathway model,

1 $$ I\leftrightarrow S\leftrightarrow N $$

the on-pathway model,

2 $$ S\leftrightarrow I\leftrightarrow N $$

and the sequential model,

3 $$ S\leftrightarrow {I}_1\leftrightarrow {I}_2\leftrightarrow \cdots \leftrightarrow N $$

where *S* corresponds to the solubilized protein, *I* to the folding intermediates, and *N* represents the native protein. However, an important aspect missing in these models is the fraction of aggregated proteins, which results from the solubilized protein or either of the folding intermediates (Kiefhaber et al. 1991). Hevehan and de Bernardez Clark described a simplified model with an on-pathway folding intermediate and off-pathway aggregation (Hevehan and de Bernardez Clark 1997) as depicted in Fig. 1.

The reaction of the solubilized protein to the folding intermediates is considered to happen immediately, simplifying the model to the off-pathway model with the two reaction rates *k*_*r*_ and *k*_*a*_ (Hevehan and de Bernardez Clark 1997). The reaction of correct folding follows a first-order reaction while the aggregation follows a reaction with an order of two or higher (Dong et al. 2004; Hevehan and de Bernardez Clark 1997; Kiefhaber et al. 1991; Zettlmeissl et al. 1979), or first order for low protein concentrations (Pan et al. 2015). In a simple dilution by batch mode refolding process, Kiefhaber et al. described the concentration of the solubilized and native protein with a second-order aggregation reaction as follows (Kiefhaber et al. 1991),

4 $$ \frac{d{c}_{SL}}{dt}=-\left({k}_r\cdotp {c}_{SL}+{k}_a\cdotp {c_{SL}}^2\right) $$

5 $$ \frac{d{c}_{NL}}{dt}={k}_r\cdotp {c}_{SL} $$

Following this scheme, an equation for the aggregated protein can be described as,

6 $$ \frac{d{c}_{AL}}{dt}={k}_a\cdotp {c_{SL}}^2 $$

with:

*c*_*SL*_: concentration of solubilized protein in the refolding vessel [g L^−1^]
*c*_*NL*_: concentration of native protein in the refolding vessel [g L^−1^]
*c*_*AL*_: concentration of aggregated protein in the refolding vessel [g L^−1^]
*k*_*r*_: reaction rate for refolding [h^−1^]
*k*_*a*_: reaction rate for aggregation [L g^−1^ h^−1^]

The refolding yield is defined as the percentage of the native protein to the total protein in the system. Due to the higher order aggregation constant, the reaction favors aggregation with higher protein concentration, thus decreasing the refolding yield. To counter the aggregation, refolding processes are diluted; however, strong dilutions are economically not feasible in large-scale production (Hevehan and de Bernardez Clark 1997). By employing analytical techniques described in “Analytical methods of refolding processes,” solubilized IBs can be determined prior to refolding through RP-HPLC while the refolding process can be monitored in real time through detecting native protein via FTIR and aggregation through DLS.

With the introduction of the dilution by fed-batch mode refolding, the existing equations are extended and the volume and denaturant concentration are included in the model (Dong et al. 2004). Due to the feeding of solubilized protein from the reservoir, the volume of the refolding vessel,

7 $$ \frac{dV_L}{dt}={F}_R $$

and the concentration of the denaturant,

8 $$ \frac{d{c}_{DL}}{dt}=\frac{F_R}{V_L}\cdotp {c}_{DR}-\frac{F_R}{V_L}\cdotp {c}_{DL} $$

change over time. Assuming the reservoir consists solely of protein in the solubilized form, Eq. 4 extends with the incoming feed and dilution,

9 $$ \frac{d{c}_{SL}}{dt}=-\left({k}_r\cdotp {c}_{SL}+{k}_a\cdotp {c_{SL}}^2\right)+\frac{F_R}{V_L}\cdotp {c}_{SR}-\frac{F_R}{V_L}\cdotp {c}_{SL} $$

Eqs. 5 and 6 extend with dilution,

10 $$ \frac{d{c}_{NL}}{dt}={k}_r\cdotp {c}_{SL}-\frac{F_R}{V_L}\cdotp {c}_{NL} $$

11 $$ \frac{d{c}_{AL}}{dt}={k}_a\cdotp {c_{SL}}^2-\frac{F_R}{V_L}\cdotp {c}_{AL} $$

with:

*V*_*L*_: volume of the refolding vessel [L]
*F*_*R*_: feed rate [L h^−1^]
*c*_*DL*_: concentration of the denaturant in the refolding vessel [g L^−1^]
*c*_*DR*_: concentration of the denaturant in the reservoir [mol L^−1^]
*c*_*SR*_: concentration of solubilized protein in the reservoir [g L^−1^]

The refolding and aggregation rates are functions of the denaturant concentration as described by Hevehan and de Bernardez Clark,

12 $$ {k}_i(t)={a}_i\cdotp {\left(1+{c}_{DL}(t)\right)}^{b_i} $$

with *i = r,a* for refolding and aggregation respectively and two modeling constants *a* and *b* (Hevehan and de Bernardez Clark 1997).

The total protein concentration in the refolding vessel,

13 $$ {c}_{PL}=\frac{F_R\cdotp t}{V_L}\cdotp {c}_{SR} $$

is dependent on the feed rate into the reactor and the concentration of solubilized protein in the reservoir and can therefore easily be calculated, assuming that no protein is present in the refolding vessel at start and the reservoir contains solubilized protein only. The refolding of proteins is on a timescale of minutes to hours (Glassey et al. 2011), if the kinetics are known the measurement frequency of corresponding PAT systems can be estimated.

Besides, other models for protein refolding have been described as well. Ryś et al. set up a more complex model including a state *M* for misfolded protein with forward and reverse reaction due to disulfide bond reshuffling (Ryś et al. 2015a, b). Moreover, further adaptation of the process model may be necessary by including additional feeds such as that of an oxidizing agent (Fazeli et al. 2011) or of a cofactor needed for protein maturation (Rogers et al. 2000) to enhance the refolding process. The control of the dO_2_ and redox potential becomes important when proteins containing disulfide bonds are processed. An oxidizing agent is necessary for the formation of disulfide bonds (De Bernardez Clark 2001) and since the reaction rates are dependent on the concentration of oxidizer and reducer (De Bernardez Clark 1998), they need to be added to the process model if they are not otherwise controlled to a fixed value (Dong et al. 2004).

Good modeling practice guidelines (Van Waveren et al. 1999; Nopens 2018) provide important steps throughout the model development to end up with an application-oriented model, such as the workflow described by Daume et al. (2020). After the formulation of equations for the reaction scheme and reaction kinetics, the model parameters, such as the folding and aggregation constants, are estimated by fitting the model to experimental data (Daume et al. 2020; Villaverde 2019). The predictive power of the model is usually determined by performing a normalized root mean square error calculation with the model simulation and experimental data.

Since the generation of a fitting process model is extremely difficult, especially in biological applications, hybrid models may offer a solution. These types of models combine the already-generated process knowledge in the form of mechanistic models with data-driven techniques such as artificial neural networks to compensate for simplifications made in the mechanistic model or to model a part of the process where mechanistic relations are unknown (Sohlberg and Jacobsen 2008).

## Monitoring of refolding processes

To monitor biological reactions, direct measurements are often hard to obtain. However, monitoring of a biological process is not solely dependent on direct measurements, but rather a combination of available direct measurements, indirect measurements, and state estimation. Direct measurements of the formation of native protein can be directly measured in real time as described in “Analytical methods of refolding processes.” For example, RP-HPLC is used to differentiate folding variants for disulfide bond containing proteins (Pathak et al. 2016), changes in the secondary structure can be analyzed with CD spectroscopy (Pathak et al. 2016), and FTIR spectroscopy (Walther et al. 2014; Pathak et al. 2016). Tertiary or quaternary protein structure can be investigated by NMR and extrinsic fluorescence (Pathak et al. 2016) and the surface charge of proteins by analysis of the zeta-potential (Pathak et al. 2016). Indirect measurements are available from soft sensors, which calculate an estimate of an otherwise unmeasurable system state by processing a measurable signal with an underlying estimation algorithm in real time (Kadlec et al. 2009; Luttmann et al. 2012). Pizarro et al. for example were able to monitor a protein refolding process with measurements of dissolved oxygen and redox potential for a disulfide bond containing protein (Pizarro et al. 2009).

Complementation of these direct and indirect measurements is realized by state estimation as a model-based approach that uses the process model to estimate the process states from system inputs and system outputs. Input for a protein refolding process in case of dilution by fed-batch mode can be the feeding rate of solubilized protein, while key performance indicators (KPIs) such as yield and space-time-yield of the refolding reaction can be the system outputs. Both KPIs can be determined online by soft sensors using the total protein concentration in the refolding vessel if the concentration of the native protein can be directly measured or accurately estimated. Simple examples of successful process estimation are based on mass, energy, or elemental balancing. Considering the law of mass conservation, unknown states can be directly calculated from others.

For more complex system descriptions, subject to non-linear reaction kinetics and internal dynamics, the most probable internal state can be estimated with non-linear Bayesian filters, such as the extended (Julier and Uhlmann 1997) and the unscented (Wan and van der Merwe 2000) Kalman filter or the particle filter (Arulampalam et al. 2002). Although some system states can be measured directly, the implementation of a state observer can be advantageous toward feedback or predictive control applications. Directly measured signals are prone to measurement errors and substantial white noise and subject to outliers, which hamper a direct use of these signals for control. A state observer may remove these artifacts in a probabilistic way, without losing the information content of the measurement.

To our knowledge, there is no state observer described specifically for the use of protein refolding. However, the aforementioned techniques are applicable, as shown in different chemical and biochemical processes (Shen et al. 2006; Sun et al. 2008; Wang et al. 2010). Furthermore, interested readers are referred to Mohd et al. for a summary of different state observers and how to design them (Mohd Ali et al. 2015).

## Control of refolding processes

Through the control of CPPs, KPIs can be held in their optimal range resulting in sufficient product quality. As a prerequisite, the CPPs (e.g., pH, temperature, agitation speed, dissolved oxygen (dO_2_), redox potential, and feeding rate) and KPIs (e.g., refolding yield, space-time yield) need to be monitored through direct measurements or soft sensors to enable their control (Kadlec et al. 2009). The concept of controllability states that a process is controllable, if every state of the system can be modified to any arbitrary value by the system inputs in finite time (Ogata 1997).

Recent advances show two approaches to achieve control of protein refolding processes. Hebbi et al. are using statistical process control and batch evolution modeling spanning from the buffer generation, over solubilization until refolding with online measurements of redox potential, pH, and temperature. These parameters are commonly measured and hence there are many probes available. Monitoring of these CPPs is sufficient to control the process in the established design space (Hebbi et al. 2019). Another approach suggests measuring the changes in the secondary structure of proteins during folding via an inline ATR-FTIR. This monitoring enables controlled termination of the refolding process instead of termination after a given reaction time, therefore preventing additional aggregation (Walther et al. 2014).

Both of these approaches offer tools to perform controlled refolding processes. However, the control in these approaches is rather data-driven than knowledge-driven. Hence, the generation of a process model is favorable. A refolding process can be controlled through its model if the model parameters describing the refolding kinetics (Eq. 12) are identifiable and every state is controllable. Model-based control techniques rely on the estimation of the system states to compare estimated with measured values and consequently drive the process toward the defined reference using appropriate control actions (Brosilow and Joseph 2002). Inversion of the model (Ferrarin et al. 2001; Kager et al. 2020) generates a control law using the feed rate of the dilution by fed-batch mode refolding as the control input to steer the refolding productivity to a constant value. A prominent model-based control technique is the model predictive control (MPC). At each time step, the actual state of the system is the initial condition for an online optimization process of state predictions based on the process model to gain the optimal system output (Grüne and Pannek 2017; Kaiser et al. 2018). The difference between the predicted output and the reference is optimized by minimizing a cost function. The user’s control strategy regarding a given protein is reflected in the cost function, where a weight is associated with both KPIs to favor one of them. Additionally, the optimization process can be subject to constraints such as physical limits for pump rates or vessel volumes (Grüne and Pannek 2017; Kaiser et al. 2018). Limitations to time-dependent changes of the feed rate as control input are important as well since they prevent an alternation between slack and high speeds.

Since MPC, in combination with soft sensors and state observers, enables the control of non-measurable process parameters, it shows better control behavior compared to other control strategies, for example elemental balance control or classical PID control (Kager et al. 2020; Ulonska et al. 2018). The prediction into the future results in faster transition behavior and better reference tracking and generates profound process knowledge (Ulonska et al. 2018). As biochemical systems are very sensitive to small changes, MPC is desirable, because the prediction of control actions in advance can prevent critical overshoots, compared to classical PID control (Kager et al. 2020).

## Proposal for a model-based refolding control strategy

With the industry gradually turning to the production of medically relevant products in the form of inclusion bodies (Humer and Spadiut 2018), there is also an increasing need for the improvement of the corresponding downstream processing methodologies that normally account for 50–80% of the manufacturing costs (Rathore et al. 2013). According to an economical assessment of various refolding strategies (Freydell et al. 2011), major cost-drivers on an industrial scale are large buffer volumes with expensive additives, requiring huge vessels and yet resulting in non-competitive yields of 15–25% of the total protein (Zhang et al. 2009) due to low recovery rates. Although other methods, such as ultrafiltration and on-column refolding, show promising results on a laboratory scale, dilution refolding is still the industrial method of choice. It is a simple, flexible, and already widely established technique with huge potential for optimization through process knowledge and control (Humer and Spadiut 2018; Linke et al. 2014; Singh et al. 2015; Vallejo and Rinas 2004). From an economical and practical point of view, increasing overall product yields of an established methodology with minimal investment is in general more favorable than the implementation of completely new techniques. Additionally, thanks to the ongoing transformation of pharmaceutical manufacturing along the QbD principles, a shift from the empirical refolding methodologies toward mechanistic knowledge-based approaches has begun. In accordance with this, several studies have been conducted pointing to the advantages of PAT in terms of increased control of refolding processes leading to better efficiencies (Hebbi et al. 2019; Humer and Spadiut 2018; Pizarro et al. 2009; Walther et al. 2014). Furthermore, IB downstream processing could become more economic by implementing continuous refolding strategies (Pan et al. 2014; Wellhoefer et al. 2014). However, to obtain the complete picture of the underlying correlations in a refolding process and establish a thorough understanding of the kinetics, an integrated approach by introducing MPC could offer a solution.

Since MPC is a versatile and useful tool for the control of a process with non-linear dynamics, we suggest the application of this technique for a protein refolding process together with the dilution by fed-batch mode method. To control the refolding process with MPC, the system needs to be modeled in such a way that every state is observable and controllable and every model parameter is identifiable from available measurements. Hence, a mechanistic process model needs to be generated, the identifiability of the model parameters must be checked, and the model parameters be estimated as described by Daume et al. (2020) and Deppe et al. (2020). We propose that such a model must be generated with a state error of below 10–15%. Furthermore, the model needs to represent the process dynamics accurately and all model parameters need to be identifiable from experimental data (Daume et al. 2020). A systematic approach for parameter estimation is presented by Brun et al., including a classification of relative parameter uncertainty to simplify this time-consuming process (Brun et al. 2002). The model described by Dong et al. (2004) can be used as the starting reaction scheme and reaction kinetics can be taken from Hevehan and Bernardez Clark (1997).

In the following, we present a proposal on a refolding control strategy: we suggest the use of two parameters, refolding yield and refolding space-time-yield, as inputs for the calculation of the optimal control strategy of the feeding rate using MPC. Both parameters can be calculated online via soft sensors. To compute the yield, the native protein concentration as well as the maximal total protein concentration in the refolding vessel must be known. The refolding yield,

14 $$ \mathrm{Refolding}\ \mathrm{yield}=\frac{c_{NL}}{c_{PL}} $$

is the quotient of native protein concentration in the refolding vessel over the total protein concentration in the refolding vessel. The refolding space-time-yield,

15 $$ \mathrm{Refolding}\ \mathrm{space}-\mathrm{time}-\mathrm{yield}=\frac{c_{NL}}{t} $$

is the amount of native protein which is produced per volume of the refolding vessel and per process time.

**Fig. 2:**
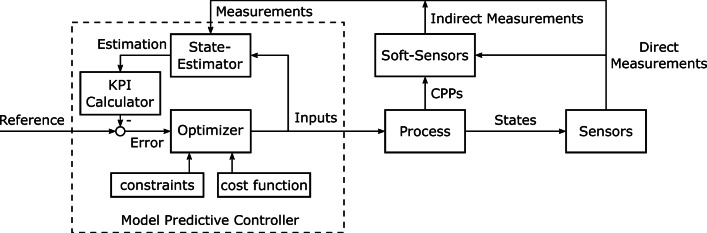
Control strategy scheme. Combined direct and indirect measurements of CPPs and states of the process are used for state estimation together with computed control inputs. The KPI yield and space-time-yield are computed from the state estimate and their error compared to the reference signal is minimized by the optimizer of the model predictive controller according to the cost function and fulfilling constraints to achieve an optimal control input for the process

Sensors and soft sensors can measure enough of the internal states of the process such that the process is completely observable and feed them to the controller (Fig. 2). After state estimation, the KPIs are calculated and the optimizer computes the optimal control inputs for the refolding process by minimizing the error between the estimated KPIs and their respective reference signal using a cost function and possibly fulfilling constraints. The cost function allows the user to weigh a KPI over another by setting these weights process-specific according to the targeted product. If the upstream process for example is expensive, higher yields are favorable; however, if the major bottleneck of the whole production process is the refolding, higher space-time-yields may be preferred.

**Fig. 3:**
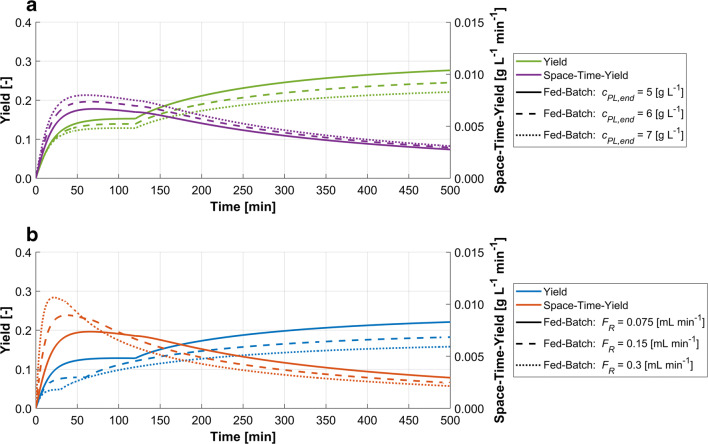
Simulations of the refolding process as dilution by fed-batch mode. After depletion of the reservoir, the refolding is continued as batch mode. **a** Influence of final total protein concentration on yield (green) and space-time-yield (purple) with constant feed rate during the fed batch. Lowest final total protein concentration (solid line) results in the highest yield and lowest space-time-yield and vice versa. **b** Influence of feed rates on yield (blue) and space-time-yield (red) with constant final total protein concentration. Lowest feed rate (solid line) results in the highest yield and lowest space-time-yield and vice versa

**Fig. 4:**
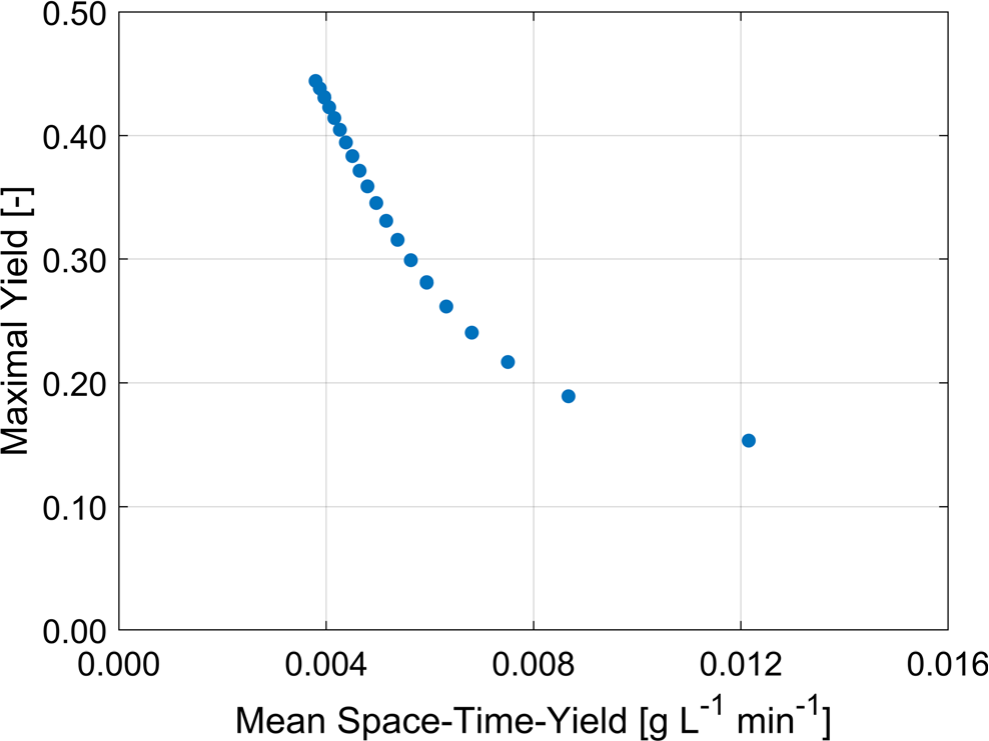
Pareto plot to illustrate the tradeoff of the maximal refolding yield against the mean space-time-yield during the fed-batch phase with 20 feed rates between 0.015 and 10 mL min^−1^ and otherwise equal conditions

The tradeoff of these two parameters is important, because focusing on a single KPI would generate misleading results. Very slow dilution rates could lead to a yield close to 100%; however, the necessary time and therefore the space-time-yield is uneconomical. Very fast feed rates on the other hand show higher productivity but decreased yield. Figure 3 visualizes the tradeoff between yield and space-time-yield based on simulations with the model and model parameters from Dong et al. (Dong et al. 2004). The solubilized protein is fed into the refolding vessel from a reservoir until it is depleted. The refolding process is continued as dilution by batch mode, during which the remaining solubilized protein folds. A comparison of varying final total protein concentration (*c*_*PL,end*_) with constant feed rate versus varying feed rates with identical final total protein concentration shows that higher yields are reached when the concentration of solubilized protein in the refolding vessel at each time step is smaller. This is achieved through either lower final total protein concentration or lower feeding rates. To further emphasize the relation between yield and space-time-yield, multiple refolding simulations by dilution in fed-batch mode with feeding rates from 0.015 to 10 mL min^−1^ are used to visualize this behavior in a Pareto plot (Fig. 4), where the maximal yield is plotted against the mean space-time-yield during the fed-batch phase.

To transform this proposal into a working refolding process with monitoring and control, the defined model needs to be parametrized and validated to ensure its predictive power and applicability. Parametrization is usually performed by applying the weighted residual sum of squares WRSS, and model validation is performed by calculating and evaluating the goodness of fit (*R*^2^) and the normalized root mean square error (NRMSE) of model simulations and experimental data (Daume et al. 2020; Deppe et al. 2020). Additionally, other methods such as Akaike information matrix (AIC) or Bayesian information criteria (BIC) give useful information about the risk of overfitting (the model fits the training data but fails with further datasets) and underfitting (approximate model is too simple to accurately predict the reaction kinetics), while incorporating the simplicity of the model in their evaluations (Deppe et al. 2020). For both parameter estimation and model validation, multiple data sets are necessary to prevent overfitting and to achieve a robust and applicable process model. Since the kinetics of the refolding process are generally understood, the model parameters can be estimated from few datasets. However, the addition of kinetic dependencies on oxidizing agent and cofactor as well as misfolded proteins and folding intermediates as states, the number of necessary datasets can rise sharply if the correct model structure is unclear.

## Conclusion

Although biopharmaceutical manufacturing has been transforming along the QbD principles in the recent years, industrial refolding is still conducted based on empirically established procedures instead of sound process knowledge. The reason behind this lies within the complex nature of these processes requiring cross-disciplinary methodology and knowledge to identify the key correlations. Until these black box processes are resolved, considerable efforts need to be made in terms of refolding process development that is costly, requires time, and yet results in non-scalable and strictly product-specific techniques with low yields.

However, the generation of mechanistic process knowledge on refolding kinetics could establish universally applicable methodologies within product families. To achieve this, real-time monitoring of relevant process variables with online sensors and soft sensors is indispensable. The analytical methods described in this review could provide a good basis for this; however, as previously discussed, the monitoring of refolding kinetics is still very challenging due to low protein concentrations. Additionally, not every process variable is directly measurable and related analytical methods have certain limitations. To meet these challenges, a modeling approach can be applied in addition to analytical techniques to estimate the non-measurable process variables in real time, thereby illuminating the complete picture of the process. Furthermore, when all relevant variables and their correlations are available, more complex model systems can be established to predict the process progression. Based on these, optimal and adaptive control trajectories are possible to achieve more efficient IB recovery processes, better product quality, and higher yields. Furthermore, in accordance with the current trends in the industry aiming at the digital transformation of manufacturing processes, an adequate software environment is needed to support this endeavor. Such a software needs to support the real-time integration and calculation of complex models, based on the acquisition of current process data and subsequently, it should be able to provide the optimized output trajectories for the manufacturing system to realize closed-loop control. Eventually, not only refolding processes can be steered, but as a next step, the complete IB downstream processing chain could be optimized.
